# Secondary surgical cytoreduction needs to be assessed taking into account surgical technique, completeness of cytoreduction, and extent of disease

**DOI:** 10.1186/s12957-020-01853-4

**Published:** 2020-05-11

**Authors:** Juan José Segura-Sampedro, Rafael Morales-Soriano, Álvaro Arjona-Sánchez, Pedro Cascales-Campos

**Affiliations:** 1 Department of General and Digestive Surgery, Son Espases University Hospital, Spain, 07010 Palma de Mallorca, Spain. Health Research Institute of the Balearic Islands (IdISBa), Palma de Mallorca, Spain. School of Medicine, University of the Balearic Islands (UIB), Palma de Mallorca, Spain; 2 Department of General and Digestive Surgery, Son Espases University Hospital, Spain, 07010 Palma de Mallorca, Spain. Health Research Institute of the Balearic Islands (IdISBa), Palma de Mallorca, Spain; 3 General & Digestive Surgery Department, University Hospital Reina Sofía, School of Medicine, University of Córdoba, Córdoba, Spain; 4 General & Digestive Surgery Department, University Hospital Virgen de la Arrixaca, School of Medicine, University of Murcia, Murcia, Spain

**Keywords:** Ovarian cancer, HIPEC, Chemotherapy, Cytoreductive surgery, Carcinomatosis

## Abstract

Recent evidence suggested that secondary surgical cytoreduction followed by chemotherapy does not result in longer overall survival in patients with platinum-sensitive recurrent ovarian cancer.

This statement is based on a phase III multicenter, randomized clinical trial that lacks a description of the surgical protocol, the surgical technique, and the surgical variables. In a study that evaluates surgical cytoreduction, it is mandatory to assess the grade of cytoreductive surgery achieved (Sugarbaker PH, Langenbeck’s Arch Surg 384:576–87, 1999), the extent of disease using PCI (Peritoneal Cancer Index), the technique itself, and the existence of a multidisciplinary approach with extensive upper abdominal procedures in experienced centers (Ren et al, BMC Cancer 15:1-12, 2015). There is evidence proving that the quality of cytoreduction (Al Rawahi et al, Cochrane Database Syst Rev 2013, 2013), the measurement of the amount of disease by PCI (Elzarkaa et al, J Gynecol Oncol 29, 2018), and a multidisciplinary approach with supramesocolic procedures (Ren et al, BMC Cancer 15:1-12, 2015) impact overall survival.

This study fails to compare chemotherapy with secondary cytoreductive surgery since, due to the lack of variables, we can assess neither the performed surgery nor its criteria. This study should not be taken into account to recommend chemotherapy alone over a surgical approach in this group of patients.

Dear Editor,

We have read with great interest the paper by Coleman et al. [1] which states that “patients with platinum-sensitive, recurrent ovarian cancer, secondary surgical cytoreduction followed by chemotherapy did not result in longer overall survival than chemotherapy alone.”

The use of hyperthermic intraperitoneal chemotherapy (HIPEC) for ovarian cancer with peritoneal dissemination is currently experiencing expansion [2]. Recently, a phase III study [3] indicated advantageous results for HIPEC after cytoreduction versus cytoreduction alone in patients treated with HIPEC after neoadjuvant chemotherapy (NACT). Furthermore, interval cytoreductive surgery plus HIPEC proved to be a cost-effective management of stage III epithelial ovarian cancer (EOC) when NACT was administered; consequently, some societies have acknowledged the applicability of HIPEC in such clinical situations [4].

We congratulate the researchers involved in this open-label, phase III multicenter, international, industry-funded, randomized clinical trial for assessing the benefits of secondary surgical cytoreduction in platinum-sensitive surgically amenable patients.

The aim of the GOG-0213 trial was double. First, to prove that bevacizumab (drug produced by the funder of the study) improves overall survival (OS) when added to paclitaxel and carboplatin chemotherapy followed by maintenance bevacizumab. Second, to prove that secondary surgical cytoreduction in platinum-sensitive surgically amenable patients improves OS compared to the proposed chemotherapy protocol.

Four hundred eighty-five patients were randomly assigned to control arm (surveillance with no surgery, *n* = 245; 240 followed this strategy, 5 patients received surgery) and to experimental arm (cytoreductive surgery, *n* = 245; 15 declined surgery, 221 were finally operated). The study failed to show evidence for improved OS in the experimental group.

Nevertheless, the study lacks a description of the surgical protocol, the surgical technique, and the surgical variables. In a study that evaluates surgical cytoreduction, it is mandatory to assess the grade of cytoreductive surgery achieved. The term used, “no gross residuum”, does not fulfill this mission. This can be achieved with the completeness of cytoreduction score (CC-score) [5], which is commonly used in peritoneal carcinomatosis, although there are different scores that could have been used as well [6]. This is important because there is evidence proving that quality of cytoreduction impacts OS [7–9]. If we do not know how complete was the cytoreduction, there is a huge bias affecting the results of the whole paper which prevents any evaluation of the cytoreductive surgery per se regarding OS.

Another fundamental parameter that should have been taken into account is the extent of disease using PCI (Peritoneal Cancer Index) [10]. It is mentioned in the paper that “more than half the patients who were considered for this trial had two or fewer sites of recurrent disease.” This is not the proper way to evaluate or to express the amount or extent of disease. An accurate description of the disease extension should have been made in both groups. There is evidence that the measurement of the amount of disease by PCI is a main prognostic factor regarding the indication of cytoreductive surgery and OS [11]. We do not know the number of patients with or without limited disease, neither the extent nor the amount in this study.

As previously stated, there is no information about the surgical protocol or the surgical team. This trial involved a large number of centers (more than 50), but at least 20 of them only managed to recruit few patients (< 10) despite a recruitment period of almost 10 years. We do not know if there was a collaborative surgical approach with a supramesocolic surgical protocol. There is evidence that a multidisciplinary approach [12] with extensive upper abdominal procedures in experienced centers [13] in ovarian cancer impacts OS. We do not know if the surgery performed by all the teams fulfilled these criteria; therefore, OS is biased again at this point.

Although “more than half the patients had two or fewer sites of recurrent disease,” only 68% had a “complete cytoreductive surgery.” These results are somehow inconsistent and may reflect the above-mentioned points: lack of a multidisciplinary approach, no supramesocolic protocol, absence of PCI evaluation, no score for completeness of cytoreduction, and lack of high-volume centers.

To conclude, this study proved that the addition of bevacizumab to standard chemotherapy, followed by maintenance therapy until progression, improved the median overall survival in patients with platinum-sensitive recurrent ovarian cancer [14]. Nevertheless, it has failed to compare this chemotherapy protocol with secondary cytoreductive surgery since, due to the lack of variables, we can assess neither the performed surgery nor its criteria. The results of this study should not be taken into account to recommend chemotherapy alone over a surgical approach in this group of patients.

## Data Availability

There is no other data to be shared.
